# Iterated Ritz and conjugate gradient methods as solvers in constrained form-finding: A comparison

**DOI:** 10.1016/j.heliyon.2021.e07011

**Published:** 2021-05-06

**Authors:** Elizabeta Šamec, Petra Gidak, Krešimir Fresl

**Affiliations:** University of Zagreb, Faculty of Civil Engineering, Zagreb, Croatia

**Keywords:** Constrained form-finding, Iterated force density method, Conjugate gradient method, Iterated Ritz method, Cable net, Gridshell

## Abstract

Constrained form-finding results in a nonlinear system of equations unless a linear form-finding method (force density method) is iteratively applied until the given constraints are satisfied. Because the goal of this paper is to contribute to the further development of this method, a brief overview of the method and its existing improvements is provided. Further improvement can be potentially expected by reducing the number of iteration steps in solving systems of linear equations in each application of the force density method. To explore this, a comparison of iterative linear solvers is conducted. The Iterated Ritz method, as a new promising solver (currently under development), was chosen for comparison with typically used conjugate gradients. Form-finding of several truss structures in both tension and compression was performed to compare the number of iteration steps necessary to obtain the solution. The presented form-finding examples indicated a significant reduction in the number of solver iteration steps, showing the potential of the Iterated Ritz method for use as a solver in linear procedures for constrained form-finding.

## 1 Introduction

**Figure 1:**
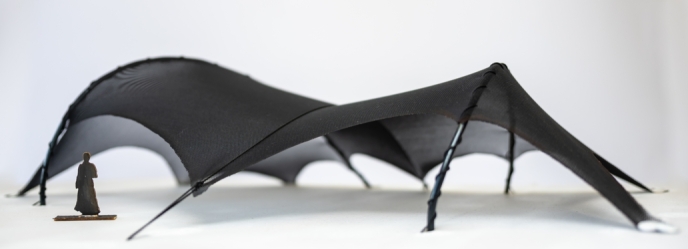
Physical model of tensile structure.

In recent years there has been a strong interest in investigating the possibilities of designing structural systems that would enable a more efficient use of resources. This is because their increased consumption affects not only the environment, but also the construction industry. Owing to the principle of gaining strength through geometry and not through materials, shape-dependent structures (such as cable nets, gridshells, shells and membranes) are a source of inspiration for various research studies aiming to provide more sustainable design and building solutions [1], [2], [3]. Advanced computational tools are needed to support such studies, because the shape of a form-active structure is not known in advance. Further, it needs to satisfy both the restrictions imposed by static equilibrium and boundary conditions, and the additional architectural and fabrication constraints. To find such a shape, we have to seek a compromise between the geometry and the forces by using a numerical process known as constrained form-finding. In the past, form-finding was not possible without using physical models (Fig. 1), because they enable an intuitive design process through interactive shape exploration. If the same experience wants to be created in a form-finding tool, real-time interventions on the geometry of the structure inside the computer-aided design tool are necessary. To develop such an interactive tool, the execution time of the implemented algorithm needs to be minimal.

The equations of the numerical model in a form-finding problem are based on large displacements; they are highly nonlinear, and in most methods do not depend on deformations, but only on the equilibrium conditions of the nodes. The number of potential algorithms to be used is quite vast [4], [5], [6]; however, despite numerous studies in the literature on form-finding methods in the last 50 years, comparison studies remain rare. Owing to the different nomenclature, mathematical structures, and notations used, it is difficult to obtain a straightforward comparison. Also, the specific implementation of each method determines its success in practical usage [7]. Therefore, only a few studies have compared the actual performance of such methods. An important contribution to the comparison of form-finding methods is given in [6], [8], where a generic form-finding method was developed to avoid at least some of the aforesaid problems. For minimal-length nets, 10 different methods were compared, and the result showed that the geometric methods seemed to be superior to the stiffness and the dynamic methods.

The basis of the category of geometric methods is the force density method (FDM), which was developed in the 1970s for form-finding of prestressed cable structures [9], [10]. The advantages of the FDM are its fast preliminary shape generation and easy exploration of various feasible solutions. The force density method emerged from a formal manipulation of mathematical expressions, as a ratio of force and length in an element. The resulting system of linear equations is easier to solve than the original nonlinear system. Therefore, a number of improvements, extensions, and modifications to this method have been proposed, e.g. [11], [12], [13]. However, as the geometry of structure gets more complicated, it becomes harder to choose the distribution of force densities in order to meet not only structural requirements but also to design a functional structure that is at the same time architecturally pleasing. Such constrained form-finding demands an extension to the nonlinear FDM. By using the nonlinear FDM, which was also introduced in [9], certain structural requirements can be satisfied: the prescribed force values in selected elements, the prescribed lengths of the elements in an equilibrium configuration, and the prescribed unstrained lengths of the elements. To avoid the nonlinear system of equations, but still attain the prescribed constraints in the elements, an iterative application of the linear FDM can be used as an alternative. For constrained form-finding, when the FDM is iteratively applied, the equation system is solved multiple times. Therefore, the standard algorithm needs to be accelerated to address the necessary speed requirements.

As tensile membrane structures and cable nets have similar properties (there are only tensile internal forces and both have fixed supports), the FDM was in [14], [15] extended to form-finding of tensile membranes, with further development in, among others, [11], [16], [17].

The modified form of the FDM was in [18] applied to cable domes and tensegrity structures, which are also related to, but in some important aspects (hereafter briefly mentioned) different from cable nets. Because tensegrity structures consist of tensile cables and compressive struts, force densities have positive or negative sign and therefore force density matrices can have negative eigenvalues. Also, tensegrities are free standing structures and accordingly, systems of force density equations are homogeneous. As a consequence, the equilibrium and the force density matrices must be rank deficient. Application of the FDM as an analytical and/or numerical form-finding tool for tensegrity structures and cable domes is further studied in [11], [12], [19], [20], [21], [22], [23]. In particular, in [19] various shape and symmetry constraints were introduced. In [21] the geometrical constraints, as formulated in [24], were incorporated into the authors' adaptive force density method [20]. Further, in [23], where symmetry and element grouping were also treated, the new formulation of the method has been introduced in which the primary variables are not the nodal coordinates but the Cartesian components (projections on coordinate axes) of cable and strut lengths.

Another “popular” method for form-finding of cable nets, membrane structures and tensegrities is the dynamic relaxation method [25], [26], [27], which was in [28] extended to constrained form-finding. It should be mentioned that form-finding problems can be “translated” to minimization problems, and then various optimization algorithms can be applied, e.g. [29]. On the other hand, the FDM has been used for the simultaneous optimization of the geometry and the topology of trusses [30].

In Section 2, an overview of the development of the Iterated FDM (IFDM) [31] and the Inexact Iterated FDM (IIFDM) [32] is presented as an introduction to the problem of creating a linear procedure for constrained form-finding that would answer the need for quick realisation in future interactive tools. In this paper further improvement, in the sense of reducing the number of iteration steps necessary to solve the system of linear equations in each iteration of the FDM, is explored. Therefore, the new solver named Iterated Ritz method (IRM) [33], [34], described in Section 3, is incorporated into the IFDM and IIFDM procedures. In Section 4, through various examples of spatial truss structures in tension and compression, its performance is compared with that of conjugate gradients (standard solver).

## 2 Iterated FDM – prior work

The starting point for form-finding of spatial truss structures in tension or compression are the nonlinear equilibrium equations of free nodes. The equilibrium equation of node *i* in direction *x* is

(1) $$\sum_{\{i,j\}\in E_{i}}S_{i,j}\;\frac{x_{j}-x_{i}}{\ell_{i,j}}\;+\;f_{i,x}\;=\;0,$$

where $E_{i}$ denotes the set of elements connected to node *i*, $S_{i,j}$ the force value in element {i,j}, $\ell_{i,j}$ is the length of that element and $f_{i,x}$ is the *x*-component of the external load acting on node *i*. Similar expressions can be written for the *y* and *z* coordinates. The force density coefficient is defined as a ratio of force and length:

(2) $$q_{i,j}=\frac{S_{i,j}}{\ell_{i,j}}.$$

As mentioned in the Introduction, this ratio can be used to linearise nonlinear equilibrium equations:

(3) $$\sum_{\{i,j\}\in E_{i}}q_{i,j}\;(x_{j}-x_{i})\;+\;f_{i,x}\;=\;0.$$

The unknowns are the coordinates of the free nodes (components of vectors **x**, **y** and **z**). These systems have the same system matrix **A**; however, they vary in vectors on the right–hand side ($x_{f}$, $y_{f}$ and $z_{f}$), the components of which are the coordinates of fixed nodes, as it can be seen in Algorithm 1. As indicated in [35] and [36], for the constrained form-finding the linear FDM can be preserved and force constraints can be partially or completely fulfilled by applying the procedure iteratively and by changing the force density values in each iteration step. Force densities are recalculated using the force or the length values from the previous iteration step:

(4) $$q_{i,j}^{\left(k\right)}\;=\;q_{i,j}^{\left(k-1\right)}\;\frac{{\bar{S}}_{i,j}}{S_{i,j}^{\left(k-1\right)}}\;\text{or}\;q_{i,j}^{\left(k\right)}\;=\;\frac{{\bar{S}}_{i,j}}{\ell_{i,j}^{\left(k-1\right)}},$$

where $q_{i,j}^{\left(k\right)}$ is the force density in the current *k*–th step, ${\overset{¯}{S}}_{i,j}$ is the target force value and $S_{i,j}^{\left(k-1\right)}$, $l_{i,j}^{\left(k-1\right)}$ are, respectively, the force and length values obtained in the previous step. The iteration continues until the difference between the obtained and the target force value is smaller than the desired tolerance $\tau_{S}$:

(5) $$e_{S}^{\left(k\right)}\;=\;\max\;|{\overset{¯}{S}}_{i,j}-S_{i,j}^{\left(k\right)}|\;<\;\tau_{S}.$$

In [37] the same procedure was used to obtain the network with uniform distribution of forces (minimal net), i.e. ${\overset{¯}{S}}_{i,j}=\overset{¯}{S}$.

**Algorithm 1:**
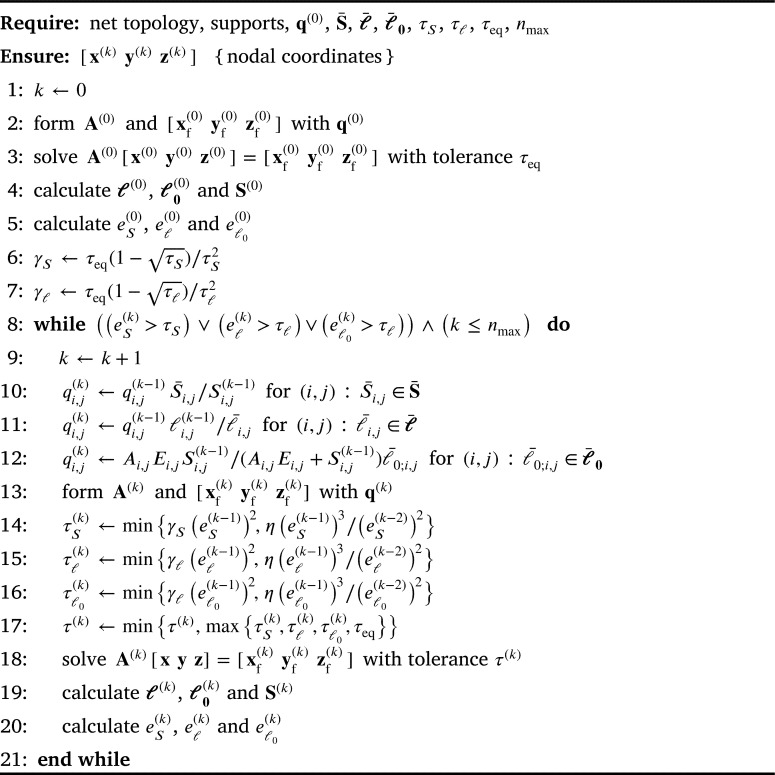
Inexact Iterated FDM algorithm.

In [31], the described procedure was extended to attain the prescribed lengths of the elements. The target element length ${\bar{\ell }}_{i,j}$ can be attained in the same way as for the forces, by recalculating the coefficients in each step using one of the following two expressions:

(6) $$q_{i,j}^{\left(k\right)}\;=\;q_{i,j}^{\left(k-1\right)}\;\frac{\ell_{i,j}^{\left(k-1\right)}}{{\overset{¯}{\ell }}_{i,j}}\;\text{or}\;q_{i,j}^{\left(k\right)}\;=\;\frac{S_{i,j}^{\left(k-1\right)}}{{\overset{¯}{\ell }}_{i,j}}.$$

Again, the iteration is terminated when further conditions are satisfied:

(7) $$e_{\ell }^{\left(k\right)}\;=\;\max\;|{\overset{¯}{\ell }}_{i,j}-\ell_{i,j}^{\left(k\right)}|\;<\;\tau_{\ell }\;\text{and/or}\;e_{S}^{\left(k\right)}\;=\;\max\;|{\overset{¯}{S}}_{i,j}-S_{i,j}^{\left(k\right)}|\;<\;\tau_{S},$$

where $\tau_{\ell }$ is the prescribed length tolerance and $\tau_{S}$ is the prescribed force tolerance. It is evident that the length constraint (6) and the force constraint (4) can not be assigned to the same element.

For the derivation of the unstrained length constraint, a relation among the force value, the strained and the unstrained length value of the element is required [9]. Assuming the linear Hooke's law of elasticity, which describes the material relationship between force and element elongation, the relation between strained and unstrained length can be defined as:

(8) $$\ell_{i,j}=\frac{A_{i,j}E_{i,j}+S_{i,j}}{A_{i,j}E_{i,j}}\;\ell_{0;i,j},$$

where $E_{i,j}$ is the modulus of elasticity and $A_{i,j}$ is the cross–sectional area of the element {i,j}. By using such element length definition in the force density expression from (6), we obtain

(9) $$q_{i,j}^{\left(k\right)}\;=\;\frac{A_{i,j}E_{i,j}S_{i,j}^{\left(k-1\right)}}{(A_{i,j}E_{i,j}+S_{i,j}^{\left(k-1\right)})\;{\overset{¯}{\ell }}_{0;i,j}}\;.$$

The force density coefficient defined in such a manner can then be used to impose the unstrained length constraint in addition to the force and length constrains from the initial set–up of the algorithm. $A_{i,j}E_{i,j}$ need not be real stiffness; it is a parameter that can be used to control the change in distance between nodes. By increasing its value we are striving towards ${\overset{¯}{\ell }}_{0;i,j}={\overset{¯}{\ell }}_{i,j}$ and the unstrained length constraint becomes the length constraint.

The proposed framework shows some advantages: it does not need an initial estimation of the solution, unlike other iterative procedures it gives an equilibrated configuration in each iteration step, and it obtains specified lengths of elements without introducing Lagrange multipliers. The last advantage is a valuable argument in favour of iteratively using the linear FDM, because length constraints are usually introduced into the calculations by using Lagrange multipliers (a nonlinear system is obtained) and the Newton–Krylov methods [38] show slow convergence in case of more complicated examples. The convergence problem can be explained by the fact that this is a saddle point problem [31].

However, the proposed procedure also sometimes requires time–consuming calculations, rendering it inappropriate for the intended interactive work. Hence, in [32] a method was proposed to reduce the number of iteration steps necessary to solve the equation system in an attempt to accelerate the procedure. To do so, in every iteration step of the IFDM (outer loop) the accuracy for system solving (inner loop) is optimised. If the computed force values or the element lengths in some iteration of the IFDM are not near the required ones, it is reasonable to solve the system only approximately, and with less accuracy the further away they are. Therefore, the tolerance for system solving is optimised to change gradually from initial tolerance towards the specified tight tolerance $\tau_{\mathrm{eq}}$ providing the final solution in equilibrium. The idea, and the name of the method, was borrowed from the inexact Newton methods introduced in [39]. A simple pseudocode for such an extended procedure, with input data and a sequence of instructions, is given in Algorithm 1.

The tolerance to achieve in system solving $\tau_{\mathrm{eq}}$ depends on the tolerances for the target values of the forces ($\tau_{S}$) and element lengths ($\tau_{\ell }$) ((5) and (7), respectively). It is known from linear algebra that a system of linear equations should be solved at least with the tolerance $\tau_{\mathrm{eq}}\;=\;\min\;(\tau_{S}/\alpha \;,\tau_{\ell }/\alpha )$, where *α* is an estimate of $\left||A^{-1}|\right|$. If system wants to be solved in the *k*–th step of the outer loop using the tolerance that will mirror the difference between the calculated values of forces or lengths and the required ones, tolerance $\tau^{\left(k\right)}$ should depend on error $e^{\left(k\right)}$. To determine the rule for calculating the tolerance in each step, the ratio of the tolerances for the inner and outer loops in the current step *k* needs to be equalized to their ratio in the final step. Since the error (in this case, the force error) is not known before the system is solved, the error value from the previous step, $e_{S}^{\left(k-1\right)}$, is used:

(10) $$\frac{\tau_{S}^{\left(k\right)}}{e_{S}^{\left(k-1\right)}}\;=\;\frac{\tau_{\mathrm{eq}}}{\tau_{S}}.$$

The relationship between the outer and the inner loop from (10) is further adjusted in [32] and the first expression in the tolerance optimisation rule (11) is obtained. The second expression is added to take into account the reduction rate of error $e_{S}^{\left(k\right)}$ by considering the value ratio in two consecutive steps (similar to that in [40]), where *η* is the constant that provides the additional control.

(11) $$\tau_{e_{S}}^{\left(k\right)}\;=\;\min\left\{\frac{\tau_{\mathrm{eq}}\;(1-\sqrt{\tau_{S}})}{\tau_{S}^{2}}\;{\left(e_{S}^{\left(k-1\right)}\right)}^{2},\;\eta \frac{{\left(e_{S}^{\left(k-1\right)}\right)}^{3}}{{\left(e_{S}^{\left(k-2\right)}\right)}^{2}}\right\}.$$

Like those for elements with force constraints, tolerance optimisation rules can be defined for elements with length and unstrained length constraints (see Algorithm 1). Among the three obtained tolerances, the smallest tolerance (the largest value) is then taken as the tolerance for solving the equation system. The tolerance in the *k*–th step should not be smaller than $\tau_{\mathrm{eq}}$ in the final step, and since the convergence of the outer loop can be nonuniform, it is necessary to verify that the tolerance obtained in the ongoing step is equal to or smaller than the tolerance obtained in the previous one. Therefore, an additional constraint is introduced:

(12) $$\tau^{\left(k\right)}\;=\;\min\;(\tau^{\left(k-1\right)},\;\max\;(\tau_{S}^{\left(k\right)},\tau_{\ell }^{\left(k\right)},\tau_{\ell_{0}}^{\left(k\right)}\tau_{\mathrm{eq}}).$$

The new procedure inherits the beneficial features of the original scheme; however, it should be noted that because the equation system is solved only approximately in nearly all steps, the obtained dispositions of nodes are not in equilibrium until the last steps are reached. The numerical examples presented in [32] and [41] have shown the beneficial effect of tolerance optimisation on a number of iteration steps needed to solve the system of equations.

## 3 Iterated Ritz method

If the tolerance for solving a system of linear equations is optimised, further iteration and (consequently) time reduction can only be expected from the solver itself. The original versions of the IFDM and IIFDM use conjugate gradient method to solve the systems of linear equations in each step of the outer loop. The potential new solver, for it to be integrated into the existing research, has to be an iterative solver with a symmetric positive definite (SPD) matrix. Therefore, the IRM – the solver proposed by the project [42], is considered to be incorporated into the IFDM and IIFDM procedures. The solver is a promising alternative to the standard or the preconditioned conjugate gradient method.

It was shown in [43] that, if Ax=b is a real linear system with an SPD matrix of order *n*, the solution can be sought through successive minimisation of the corresponding energy functional or the quadratic form

(13) $$f(x)\;=\;\frac{1}{2}\;x^{T}A\;x\;-\;x^{T}b$$

inside a small subspace formed at each iteration. An iterative procedure is usually defined as

(14) $$x^{\left(k+1\right)}=x^{\left(k\right)}+p^{\left(k\right)},$$

where, according to [33], [34], the solution increment $p^{\left(k\right)}$ is a linear combination of a suitably generated set of linearly independent coordinate vectors $\Phi^{\left(k\right)}=[\;\phi_{1}^{\left(k\right)}\;\;\phi_{2}^{\left(k\right)}\;\ldots \;\phi_{m}^{\left(k\right)}\;]$ spanning a subspace in the current step:

(15) $$p^{\left(k\right)}\;=\;\Phi^{\left(k\right)}a^{\left(k\right)},$$

where $a^{\left(k\right)}$ is a vector of (unknown) coefficients. A local minimum is sought within the subspace, thereby decreasing the total energy of the system, which eventually converges to the required minimum.

The energy decrement can also be expressed as a quadratic function:

(16) $$\Delta f(a^{\left(k\right)})\;=\;\frac{1}{2}\;{\left(a^{\left(k\right)}\right)}^{T}\;{\overset{¯}{A}}^{\left(k\right)}\;a^{\left(k\right)}\;-\;{\left(a^{\left(k\right)}\right)}^{T}\;{\overset{¯}{r}}^{\left(k\right)},$$

where ${\overset{¯}{A}}^{\left(k\right)}={\left(\Phi^{\left(k\right)}\right)}^{T}\;A\;\Phi^{\left(k\right)}$ is the SPD generalised (Ritz) matrix and ${\overset{¯}{r}}^{\left(k\right)}={\left(\Phi^{\left(k\right)}\right)}^{T}\;r^{\left(k\right)}$ is the generalised residual vector, with a common definition of the residual $r^{\left(k\right)}=b-Ax^{\left(k\right)}$. When (16) is minimised, the following system of equations is obtained:

(17) $${\overset{¯}{A}}^{\left(k\right)}a^{\left(k\right)}\;=\;{\overset{¯}{r}}^{\left(k\right)}.$$

By solving this, using any direct method, we obtain the solution necessary to determine the solution increment in (15) and then the approximate solution in (14) can be updated. The subspace dimension *m* is much smaller than the number of unknowns, to have an iteration step that is the fastest possible. The main problem is, therefore, how to generate a small and efficient subspace in a short time, so that the energy depletion per step is the largest possible and the number of steps is vastly reduced. A simple pseudocode is given in Algorithm 2 to summarise the IRM procedure.

**Algorithm 2:**
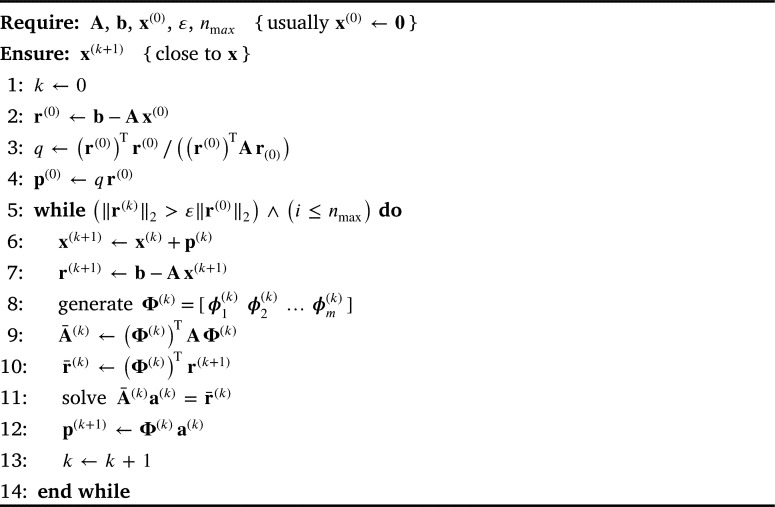
IRM algorithm.

The solver showed good initial results in [33], [34] for extremely large systems (10^7^ unknowns) with sparse matrices and therefore its performance in case of systems with fewer unknowns but solved in a large number of iteration steps is worth investigating hereafter.

## 4 Use of IRM in Iterated FDM and Inexact Iterated FDM

As explained in the earlier chapter, to try to reduce the number of iteration steps, instead of the conjugate gradient method, the IRM can be used.

To compare the number of steps necessary to solve the systems of equations using different methods, we performed form–finding of several spatial truss structures in tension and compression. First, the cable nets are analysed, with an idea to use characteristic examples of the following groups: the minimal net with fixed edges, the net with edge cables containing the force and the length constraints and the high–point net with a loop containing all three types of constraints. At the end, the form-finding of a stadium roof is presented as an example of a gridshell.

### 4.1 Cable nets

In form-finding of cable nets, free nodes are acted upon only by the prestressing forces in connected elements, i.e. $f_{i,x}$ in the equation (3) is equal to zero.

In the first example all cables have fixed supports and their height is determined by the expression:

(18) $$z\;=\;\frac{a}{2}\;\ln\frac{\cos\;(x/a)}{\cos\;(y/a)}.$$

**Figure 2:**
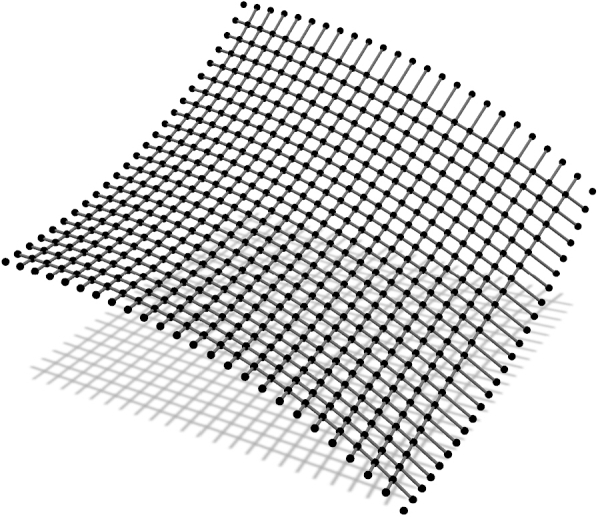
Net with “rigid” supports.

The network covers a ground area ${\left[0,a\right]}^{2}$ with 46 cables interesting in 529 nodes (Fig. 2). Therefore, 529 equations for each of the three coordinate directions need to be solved in every step of the outer loop. The minimal net is sought, with unit force values in all cables.

**Figure 3:**
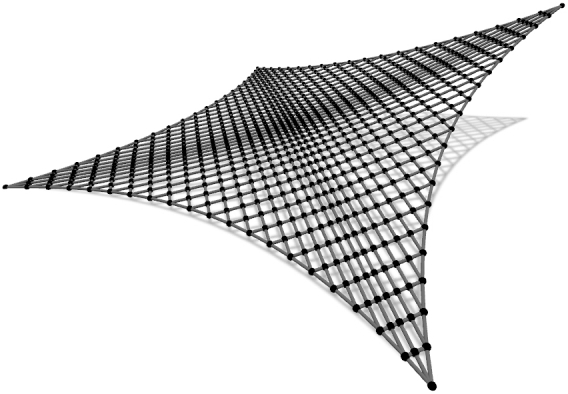
Net with edge cables.

The second net consists from 78 inner cables and four boundary cables. The three corner points lie in the horizontal plane and the fourth one is elevated (Fig. 3). In the inner cable elements forces are specified, while for the boundary elements lengths are assigned. New coordinates are determined for 837 free nodes.

**Figure 4:**
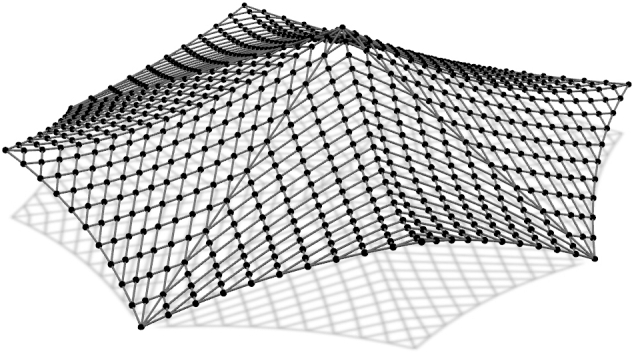
Net over octagon.

The third example has nine anchor points. Boundary cables connect eight supports that lie in the horizontal plane while the “ridge” and “valley” cables connect those supports with a high anchor (Fig. 4). For the elements of the boundary cables lengths are specified. The same is done for the elements of the “ridge” and “valley” cables, while in other cables the force values are specified.

**Figure 5:**
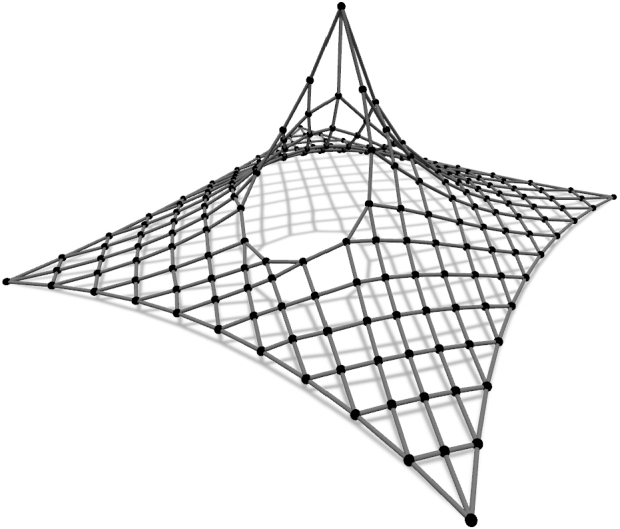
Net with internal loop.

The forth net is a high point net with an internal loop (Fig. 5). The net is spread over a square ground-plan area with four corner supports and one inner high point support. In this case, form-finding procedure with the force constraints on the inner cables and the length constraints for the elements of the edge cables as well as the loop, shows sliding of the cables. To prevent cable sliding, the unstrained length constraints are used.

Besides preventing sliding, the goal is to keep the distribution of the prestressing force quite uniform. The force distribution and net deformation resulting from the two different design approaches were compared in [41]. In the first design approach (Net 4.1), the unstrained length constraints were assigned to all internal elements in order to obtain an equidistant net. In the second design approach (Net 4.2), the unstrained lengths were assigned locally, while for the rest of the elements of the inner cables, the force constraints were assigned to control the force distribution.

The main characteristics of the cable nets, the constraints and the prescribed tolerances are given in Table 1. The constant in tolerance optimisation is η=0.02 and for the nets 4.1 and 4.2 the value of $E_{i,j}A_{i,j}$ is defined as 100 for all cables.

**Table 1:** Net characteristics.

	Free nodes	Elements	Force constr.	Length constr.	Unstrained length constr.	*τ*_*S*_	*τ*_*ℓ*_	*τ*_eq_
Net 1	529	1 104	1 104	−	−	10^−4^	−	5 ⋅ 10^−7^
Net 2	837	1 680	1 600	80	−	10^−4^	10^−4^	5 ⋅ 10^−7^
Net 3	832	1 720	1 520	200	−	10^−4^	10^−3^	5 ⋅ 10^−7^
Net 4.1	208	422	−	52	370	−	10^−3^	5 ⋅ 10^−6^
Net 4.2	208	422	322	52	48	10^−4^	10^−4^	5 ⋅ 10^−7^

The numbers of steps necessary to find the equilibrium shape of presented cable net structures using the Iterated FDM are given in Table 2, and Table 3 depicts the analogous numbers for the Inexact Iterated FDM. The number of steps in the outer loops is the number of the solved systems equations, or in other words, the number of the net configurations calculated by the algorithm when approaching the solution that satisfies the set requirements with the prescribed tolerances. This number does not depend on the solver used because the net configuration and the resulting system of equations are interdependent. The force density coefficients for the system in a certain step are calculated according to the expression (4) and/or (6), by using the nodal coordinates from the configuration in the previous step. Because the input on initial force density distribution is the same no matter the solver, the formed systems of equations are very similar throughout the computation. The negligible differences between the numbers for outer loop steps are result of the rounding errors characteristic to each solver. Our goal is therefore set on reducing the number of steps in the inner loops, because this number is the sum of steps necessary to solve the three linear systems in every form-finding step (the outer loop).

**Table 2:** Comparison of the conjugate gradient method and the IRM according to the number of steps in the outer and the inner loops of the Iterated FDM for form-finding of cable nets.

Iterated FDM							

		CG	IRM (2)	IRM (3)	IRM (4)	IRM (5)	IRM (6)

Net 1	Outer	395	395	395	395	395	395
	Inner	11 815	11 803	4 993	3 599	3 062	2 804

Net 2	Outer	872	885	898	863	846	852
	Inner	45 626	46 259	12 631	8 147	6 799	6 161

Net 3	Outer	299	300	304	296	295	297
	Inner	24 844	24 852	6 204	3 741	2 926	2 490

Net 4.1	Outer	3 424	3 408	3 405	3 467	3 483	3 417
	Inner	46 533	46 007	19 546	15 268	12 959	11 654

Net 4.2	Outer	305	304	306	302	305	305
	Inner	17 239	17 233	5 656	3 443	2 835	2 508

**Table 3:** Comparison of the conjugate gradient method and the IRM according to the number of steps in the outer and the inner loops of the Inexact Iterated FDM for form-finding of cable nets.

Inexact Iterated FDM							

		CG	IRM (2)	IRM (3)	IRM (4)	IRM (5)	IRM (6)

Net 1	Outer	395	394	394	393	395	396
	Inner	5 601	5 602	2 958	2 567	2 474	2 439

Net 2	Outer	918	897	878	845	866	862
	Inner	20 455	18 682	7 304	5 524	5 284	5 059

Net 3	Outer	267	272	282	286	306	308
	Inner	7 180	7 278	2 672	2 249	1 935	1 822

Net 4.1	Outer	3 413	3 407	3 457	3 475	3 482	3 398
	Inner	20 024	20 161	12 414	10 149	8 739	8 143

Net 4.2	Outer	301	301	293	309	311	311
	Inner	5 821	5 773	2 233	1 887	1 788	1 739

The number *n* in the IRM (*n*) denotes the number of the coordinate vectors used. The used set of coordinate vectors is similar to the one used in [34]. The first two coordinate vectors, the $\phi_{0}^{\left(i\right)}$ and the $\phi_{1}^{\left(i\right)}$, are the previous displacement increment $p^{\left(i-1\right)}$ and the current residual $r^{\left(i\right)}$. The third vector is defined by the expression $\phi_{2}^{\left(i\right)}=L^{-1}U^{-1}r^{\left(i\right)}=L^{-1}U^{-1}\phi_{1}^{\left(i\right)}$, while from the fourth vector onwards the vectors are defined as $\phi_{k}^{\left(i\right)}=L^{-1}U^{-1}A\phi_{k-1}^{\left(i\right)},\;k=3,4,\ldots$, where $L^{-1}$ and $U^{-1}$ denote the forward and the backward passes of the Gauss–Seidel iteration (i.e. one pass of symmetric Gauss–Seidel iteration).

### 4.2 Gridshells

The iterative application of the FDM is not reserved only for form-finding of prestressed cable nets. By using the tension–compression analogy [44], it can also be used for form-finding of structures that primarily consist of compression elements.

Now, the $f_{i,x}$ in the equation (3) is not equal to zero, because between the input parameters there are concentrated forces that act on free nodes of a gridshell and the unit weights of elements. The additional vertical loads are calculated by summing the half weights of the elements connected to the same node. If the target lengths of the elements are given, they are used to calculate the weights at the beginning of form-finding. Otherwise, in each iteration the weights are recalculated, according to the element lengths from the current net configuration.

The structural optimization process based on the tension–compression analogy that is applied to the design of the roof for the new stadium Kantrida in Rijeka, Croatia is described in [45], [46]. The initial form of the gridshell, given by the architect, required excessive dimensions of all steel profiles to satisfy ultimate and serviceability limit states. This is the repercussion of the irregular roof geometry, the large opening in the centre, and the significant roof span. Therefore, an optimisation procedure was needed to find a form of a gridshell with better structural efficiency. The three obtained solutions, discussed in [45], [46], are used here to test the behaviour of presented solvers during the form-finding of gridshells.

The gridshell has a tension reinforced prestressed ring at the bottom, so in the form-finding process the nodes on the bottom ring are assumed fixed. The resulting geometry of the structure is a result of specified constraints and therefore the possible geometries were sought by authors in [45], [46] using diverse constraints. For the first set of kinematic constrains, the grid A, the target force in the inner ring was set to 2100 kN and in the other elements as 150 kN (Fig. 7). In the grid B, the final geometry consists of elements of the same length (6.40 m) while for the elements of the inner ring the axial force was set to 2000 kN (Fig. 8). The equilibrium geometry of the grid A and grid B is about the same height (both are quite low). Because of the same (and relatively high) axial force in the inner ring elements of the grid A, the ring tends to a circle shape and pulls other elements towards the plan centre, what differs considerably from the initial design. At the same time, accumulation of elements at the corners is avoided which is not the case for the grid B, where accumulation results in (certain) small length of the inner ring elements (causing aesthetic and production difficulties). Hence, both grids were discarded, and another set of constraints was defined.

In the grid C, the lengths of all elements were set as kinematic constraints. In the final geometry, not all elements might have exclusively compression forces since this is sometimes impossible to accomplish. As the process of form-finding is based on tension–compression analogy, these are the tension forces and the occurrence of compression forces should be avoided. Therefore, when the force changes from tension to compression (actually, when the element force tends to zero i.e., numerically, when the force value is less than some predefined value), the constraint on the length of the corresponding element is removed.

**Figure 6:**
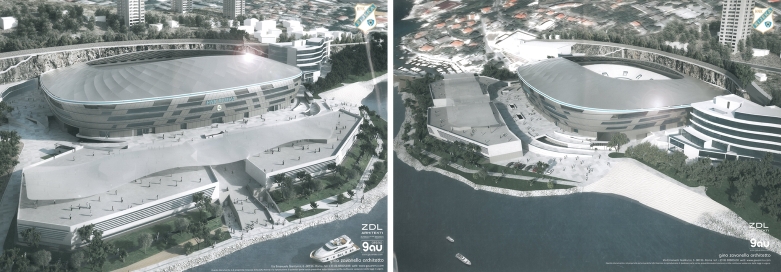
New stadium Kantrida in Rijeka: architectural renderings (Courtesy of ZDL Architects (Rijeka, Croatia) and GAUarena (Rome, Italy).)

The resulting geometry was suitable from aesthetic and functional aspects (Figs. 9 and 6). The major differences, with the respect to the original design, were the shape of the inner ring, which is no longer in the plane, and the increase of the equilibrium geometry curvature (Fig. 10).

**Figure 7:**
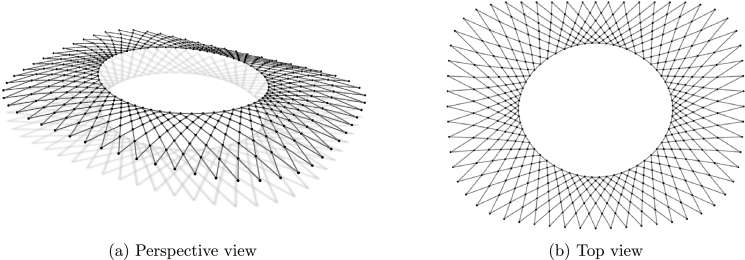
Grid A.

**Figure 8:**
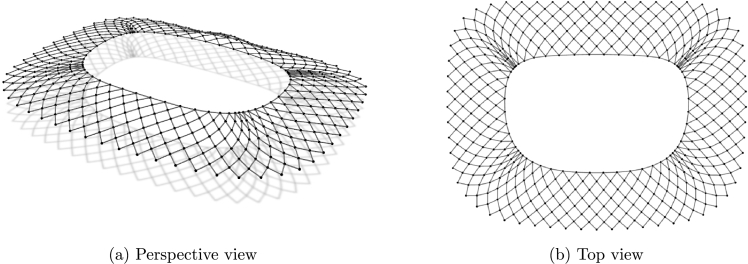
Grid B.

**Figure 9:**
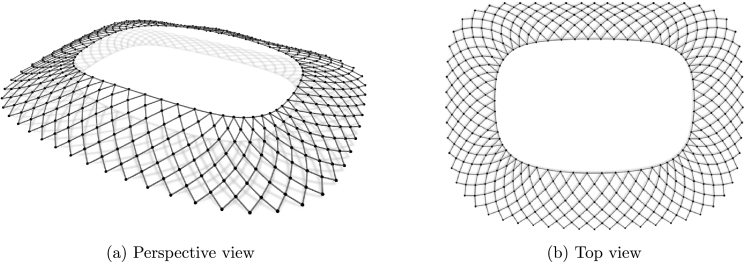
Grid C - final solution.

**Figure 10:**
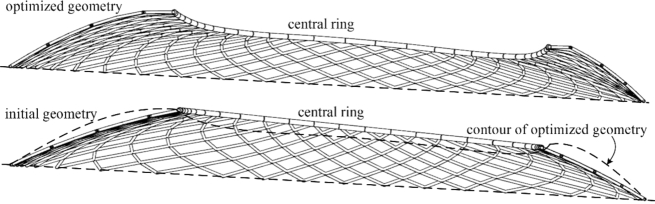
New stadium Kantrida in Rijeka: comparison of the initial and the optimized geometry.

The constraints and the other data for form-finding are given in Table 4. The comparisons of the numbers of steps for the IRM used in the IFDM an IIFDM algorithm against the conjugate gradients are given in Tables 5 and 6, respectively. For the IRM, the same set of coordinate vectors was used as in the previous cable net examples.

**Table 4:** Grid characteristics.

	Free nodes	Elements	Force constraints	Length constraints	*τ*_*S*_	*τ*_*ℓ*_	*τ*_eq_	*η*
Grid A	420	900	900		10^−3^		5 ⋅ 10^−6^	0.005
Grid B	420	900	60	840	10^−2^	10^−2^	5 ⋅ 10^−6^	0.005
Grid C	420	900		900		10^−2^	10^−5^	0.01

**Table 5:** Comparison of the conjugate gradient method and the IRM according to the number of steps in the outer and the inner loops of the Iterated FDM for form-finding of gridshells.

Iterated FDM							

		CG	IRM (2)	IRM (3)	IRM (4)	IRM (5)	IRM (6)

Grid A	Outer	1 603	1 603	1 549	1 578	1 544	1 571
	Inner	49 082	49 492	14 320	10 574	9 313	8 762

Grid B	Outer	1 536	1 528	1 554	1 521	1 524	1 526
	Inner	39 490	40 511	12 097	9 196	8 397	7 990

Grid C	Outer	7 943	7 942	7 943	7 944	7 945	7 946
	Inner	49 667	49 514	20 975	17 170	16 088	15 501

**Table 6:** Comparison of the conjugate gradient method and the IRM according to the number of steps in the outer and the inner loops of the Inexact Iterated FDM for form-finding of gridshells.

Inexact Iterated FDM							

		CG	IRM (2)	IRM (3)	IRM (4)	IRM (5)	IRM (6)

Grid A	Outer	1 648	1 659	1 531	1 629	1 639	1 545
	Inner	34 963	36 870	13 189	9 868	8 958	7 816

Grid B	Outer	1 484	1 588	1 773	1 640	1 556	1 666
	Inner	16 108	14 820	7 118	6 005	5 243	5 565

Grid C	Outer	7 966	7 968	7 941	7 941	7 945	7 940
	Inner	34 991	35 544	16 526	13 876	13 528	13 070

## 5 Concluding remarks

The Iterated Ritz method is a new linear solver intended for (extremely) large systems of equations. Efficiency is not that important for smaller systems, because all solvers have sufficiently short execution times. However, in the iterative application of the force density method linear systems are solved in each iteration step and the time spent in solving them again becomes important, particularly if the method is used in an interactive form-finding environment.

The presented comparison of the solvers, the conjugate gradients (standard solver) and the newly implemented Iterated Ritz method, allows new insights into possibilities of reducing the number of steps necessary to solve the equation systems and consequently, the computational time for a linear procedure for constrained form-finding.

Through various examples of cable nets and gridshells in Section 4, the following observations were made:

• It is possible to reduce the number of solver iteration steps in each iteration of a form-finding procedure by using the Iterated Ritz method.
• Regarding the number of steps, for two coordinate vectors the Iterated Ritz method is equivalent to the conjugate gradient method and therefore no improvement can be expected.
• Considerable reduction in the number of steps in inner loops ensues from the addition of the third coordinate vector.
• Although additions of new coordinate vectors result in further reductions, in most cases, after the fourth vector, the reduction is not significant.

As additional insides, two bottlenecks are discovered, due to which a further reduction in the execution time is not as perceivable. The first bottleneck is the generation of coordinate vectors in line 8 in Algorithm 2, and the second one is the matrix—matrix product in line 9. Considering all mentioned, the following general conclusions can be made:

• One step of the Iterated Ritz method is slower than the one step of the conjugate gradient method.
• From the third coordinate vector onwards, the generation of coordinate vectors is rather time-consuming, because the additional matrix–vector multiplications overweight the benefits from added vectors.
• Due to a significant reduction in solver iteration steps, the authors believe that the Iterated Ritz method had partially fulfilled the set goals and has a potential to become a solver in the form-finding problems if further remarks could be addressed by the future research.

It should be taken into account that the results for solver comparisons are obtained by using generic coordinate vectors, applicable to various set of problems. As pointed out by the authors in [33], [34], the key to the success of the Iterated Ritz method is the choice of subspaces in which, in every step, the solution increment is sought. The procedure for determination of the coordinate vectors, which are tailored to the problem of form-finding and the force density method, should be therefore established. Also, the possibility of directly forming the matrix ${\overset{¯}{A}}^{\left(k\right)}$ (line 9 of Algorithm 2) in some subspaces, without matrix–matrix multiplication (similar to [47]) should be investigated.

## Declarations

### Author contribution statement

Elizabeta Šamec: Performed the experiments; Analyzed and interpreted the data; Wrote the paper.

Petra Gidak: Analyzed and interpreted the data; Wrote the paper.

Krešimir Fresl: Conceived and designed the experiments.

### Funding statement

This work was supported by 10.13039/501100004488Hrvatska Zaklada za Znanost (IP-2014-09-2899).

### Data availability statement

Data will be made available on request.

### Declaration of interests statement

The authors declare no conflict of interest.

### Additional information

No additional information is available for this paper.
